# An empirical Bayesian approach for model-based inference of cellular signaling networks

**DOI:** 10.1186/1471-2105-10-371

**Published:** 2009-11-09

**Authors:** David J Klinke

**Affiliations:** 1 Department of Chemical Engineering, West Virginia University, Morgantown, WV 26506-6102, USA; 2 Department of Microbiology, Immunology & Cell Biology; West Virginia University, Morgantown, WV 26506-6102, USA

## Abstract

**Background:**

A common challenge in systems biology is to infer mechanistic descriptions of biological process given limited observations of a biological system. Mathematical models are frequently used to represent a belief about the causal relationships among proteins within a signaling network. Bayesian methods provide an attractive framework for inferring the validity of those beliefs in the context of the available data. However, efficient sampling of high-dimensional parameter space and appropriate convergence criteria provide barriers for implementing an empirical Bayesian approach. The objective of this study was to apply an Adaptive Markov chain Monte Carlo technique to a typical study of cellular signaling pathways.

**Results:**

As an illustrative example, a kinetic model for the early signaling events associated with the epidermal growth factor (EGF) signaling network was calibrated against dynamic measurements observed in primary rat hepatocytes. A convergence criterion, based upon the Gelman-Rubin potential scale reduction factor, was applied to the model predictions. The posterior distributions of the parameters exhibited complicated structure, including significant covariance between specific parameters and a broad range of variance among the parameters. The model predictions, in contrast, were narrowly distributed and were used to identify areas of agreement among a collection of experimental studies.

**Conclusion:**

In summary, an empirical Bayesian approach was developed for inferring the confidence that one can place in a particular model that describes signal transduction mechanisms and for inferring inconsistencies in experimental measurements.

## Background

Cellular response to extracellular stimuli is governed by biochemical reactions that allow the transfer of information from the cell membrane to the nucleus and back [1]. Based upon well-crafted bench experiments, many of the molecular players in the various signaling pathways are known. However, the roles that individual proteins play at specific points in time and in particular systems are largely unknown [2]. It is precisely in this situation that mathematical models are most helpful [3].

Mathematical models are tools used to rationalize about the intracellular signaling mechanisms that underpin biological response [4]. Mathematical models that describe biochemical kinetics are explicit statements about molecular-molecular interactions that are presumed to be important in a system and the corresponding dynamics of these interactions. These interactions give rise to a flow of molecular information in the form of reaction pathways [5]. The primary goal of the analysis of these reaction pathways is to make predictions: what do we expect to happen in a particular reacting mixture under particular reaction conditions, given our current understanding of molecular interactions? Similarities confirm our explicit statements while differences between the expected behaviors and new data highlight areas of uncertainty in our understanding and provide the engine for scientific progress [6]. Analogous to experimental studies, the ability of a particular mathematical model to describe a system of interest must include a statement of belief.

Belief derived from a mathematical model is expressed commonly in terms of a single point estimate for the predictions, obtained from the set of parameters that minimizes the variance between model and data [7]. Given that a model constrains the set of possible states of the system, it would be particularly valuable to provide an estimate of the uncertainty associated with the model predictions given the available data. A Bayesian view of statistics is a mathematical expression of our beliefs [8]. Beliefs are established based upon the observation of data and the interpretation of that data within the context of our prior knowledge [6]. Mathematical models provide a quantitative framework for representing prior knowledge of the detailed biochemical interactions that comprise a signaling network. The unknown parameters of the model can be calibrated against the observed network dynamics. With recent advances in computational power, computationally-intensive Bayesian techniques, such as Markov Chain Monte Carlo (MCMC) algorithms [9], are an attractive option for assessing the uncertainty in the model parameters given the calibration data and are increasingly applied to biological systems [10].

MCMC techniques provide random walks in parameter space whereby successive steps are weighted by the likelihood of observing data given the corresponding parameter values. A prior distribution is used to determine the size of proposed steps (i.e., the proposal distribution) within parameter space. The historical challenge with implementing a Bayesian approach is to specify a prior distribution for the values of the unknown parameters [8]. Conventional application of Bayesian techniques to cellular signaling networks focus on using proposal distributions that dynamically scale the prior distributions during the simulation (e.g., [11-13]), whereby the structure of the proposal distribution is unchanged during the simulation, or that is based upon the prior information encoded within the model and the parameter values at local optima (e.g., [14,15]). Moreover, a common approach in the field is to use small models as a vehicle to demonstrate new Bayesian algorithms (e.g., [11-14,16]). While these small models, sometimes called toy models, are computationally attractive, larger models foreshadow potential problems that may arise when a Bayesian technique is used in practice, such as computational efficiency. The computational efficiency of a MCMC algorithm depends highly on the structure of the proposal distribution. One of the recent advances in the field has been has been a technique that allows the MCMC algorithm to "learn" a better structure for the proposal distribution "on-the-fly." These algorithms are "empirical" Bayesian techniques in the sense that the structure of the proposal distribution is conditioned on the observed data. Adaptive MCMC (AMCMC) algorithms (e.g., [17]) dynamically adjust the proposal distribution from a non-informative prior distribution at the start of the simulation to a proposal distribution that reflects the structure in the cumulative Markov chain. The objective of this study was to develop and apply an Adaptive MCMC technique to a typical study of cellular signaling pathways. As an illustrative example, a kinetic model for the early signaling events associated with one receptor of the epidermal growth factor (EGF) signaling network was calibrated against dynamic measurements observed in primary rat hepatocytes.

## Methods

### A model for early ErbB1 signaling

To illustrate the empirical Bayesian approach, a mathematical model was developed for describing the early signaling events via the ErbB1 receptor. Developing this model was divided into three steps: specifying the model topology, calibrating the model, and estimating the uncertainty in the model predictions. In the following sections, each of these aspects is described in greater detail.

#### Model topology

**Figure 1:**
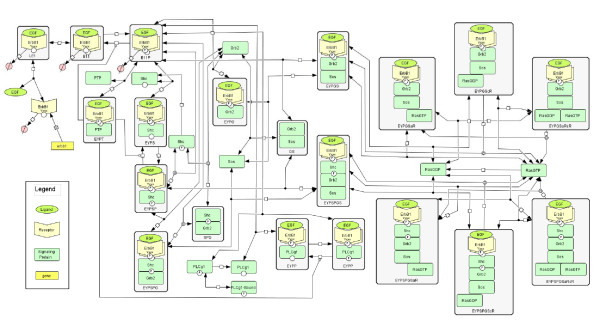
**Schematic diagram of model developed for early EGF signaling**. A schematic diagram of the biochemical events represented in the mathematical model represented using Systems Biology Graphical Notation [57]. The model represents ErbB1 synthesis and degradation; binding to EGF ligand; receptor dimerization and activation; activation of the signaling pathways associated with the binary interactions between ErbB1 + PLC*γ*-1, ErbB1 + Shc, Shc + Grb2, ErbB1 + Grb2, and Grb2 + Sos; deactivation following PTP + ErbB1 interactions; ligand-induced downregulation of ErbB1; Ras activation by ErbB1-bound Sos; and dissociation of active Shc.

The application of quantitative methods to the EGF signaling pathway was pioneered by Kholodenko et al. [18], and extended by many others (e.g., [19-22]). The model used here is similar to the original Kholodenko model but incorporates three key changes. First, the reactions were grouped into reaction classes that are defined based upon peptide motif-motif interactions [23]. Organizing cell signaling reactions into a small set of reaction classes provides a direct link to the automatic reaction network generation literature (e.g., [21,24,25]). Parameters for specific reactions were assigned based upon membership in a particular reaction class. Second, binding between proteins, which include multiple peptide motifs, was assumed competitive. In practice, this additional constraint for ErbB1 converts a differential equation for unoccupied phosphotyrosine ErbB1 (*EY P*) into an algebraic conservation equation. Finally, the molecular mechanism associated with Ras activation was modified to reflect recent biochemical observations that the activity of membrane-bound Sos is modulated via an allosteric mechanism [26]. For brevity, the resulting reaction network is shown schematically in Figure 1 and the corresponding equations can be found in an additional file (see Additional file 1). With the addition of the Ras mechanism and other modification, the resulting model, comprised of 35 non-linear ordinary differential equations and one algebraic equation, was slightly more complicated than the original Kholodenko model that contained 22 non-linear ordinary differential equations.

#### Model calibration

The resulting biochemical kinetic reaction network specifies a particular causal connectivity diagram between proteins (i.e., a set of coupled non-linear differential algebraic equations). This connectivity diagram must be coupled with parameters for each reaction to simulate the concentration changes with time. The values for the parameters were determined to be consistent with observed experimental data. In particular, dissociation constants for particular protein-protein interactions were constrained by the recent observations by MacBeath and coworkers [27,28]. In addition, measurable interaction strengths between peptide motifs were used to estimate reaction path degeneracy (Klinke DJ: Signal Transduction Networks in Cancer: Quantitative Parameters Influence Network Topology, submitted) (see Additional file 1: Table S5 for a list of the dissociation constants and values for reaction path degeneracy). These data helped reduce the number of parameters determined from 43 (38 kinetic parameters and 5 initial concentrations) in [18] to 34 (28 kinetic parameters and 6 initial concentrations) in this study.

As cellular context may influence the strength of a particular pathway [29], experimental data was extracted from the literature to calibrate the initial signaling events in a specific system: the response of primary rat hepatocytes to 20 nM of EGF. Parameter values were chosen based on appropriate measurements in rat hepatocytes if available. The changes in phosphorylated ErbB1 [18,30,31], Grb2 binding to ErbB1 [18], Grb2 binding to Shc [18], phosphorylated Shc [18,22,31], phosphorylated PLC*γ*-1 [31], Sos binding to ErbB1 [30], and the active fraction of Ras (RasGTP) [30] were calibrated to observations in isolated hepatocytes from Harlan Sprague Dawley rats upon exposure to 20 nM EGF. Initial expression of ErbB1 prior to EGF stimulation was observed to be 100 nM. However, in the absence of specific kinetic measurements in rat hepatocytes, data obtained from human cell lines were used. Total ErbB1 receptor expression was calibrated to changes in ErbB1 expression in HMEC 184A1 cells observed following exposure to 20 nM EGF [20]. In addition, the dynamics of Shc phosphorylation was also compared against measurements obtained using 10 nM EGF in the MCF-7 cell line [22]. The model equations were encoded and evaluated in MATLAB V7.0 (The MathWorks, Natick, MA). Summed squared error between experimental and simulated measurements was used to determine goodness-of-fit. Using the set of coupled differential algebraic equations, the maximum likelihood estimates for unknown parameter values were determined from these experimental data using a simulated annealing optimization algorithm [32]. The optimum values were used as a starting point for the Markov Chain.

### A Bayesian perspective on model-based inference

The expected value for some property of a model can be calculated from the following integral:(1)

where *f*(Θ) is a generic function of the model parameters. The expected value is dependent on the particular formulation of the model, *M*, and the data used in calibrating the model, *Y*. As not all combinations of parameters provide realistic simulations, values for *f*(Θ) are weighted by distribution of parameters given *M *and *Y *(i.e., the posterior distribution *P*(Θ|*M, Y*)). Using Bayes theorem, the posterior distribution of the parameters can be re-expressed in terms of more computationally tractable quantities:(2)

In equation 2, *P*(Θ|*M*) is the probability of sampling a point in parameter space Θ prior to any knowledge about data *Y *(i.e., the prior for Θ), *P*(*Y*) is called conventionally the evidence for a model, and *P*(*Y*|Θ, *M*) is the conditional probability of simulating data *Y *given Θ and *M *(i.e., the likelihood of *Y *given Θ and *M*).

As the evidence for a model can be considered as a constant, the posterior distribution of the parameters is proportional to the product of the likelihood of the data and the priors. A form of the likelihood can be obtained by assuming that the errors associated with each experiment are independent samples from multivariate normal distribution with a mean of zero:(3)

where *v *is the the dispersion matrix, *k *is the number of experimentally observed variables acquired *N*_*obs *_times, and Σ is the variance-covariance matrix of the error associated with the experimental observations. The dispersion matrix is a *k *× *k *positive-definite matrix where each element,(4)

corresponds to the normalized sum of the product of the deviation between specific observations, *Y*_*iu*_, and their respective model prediction, *M*_*i*_(Θ). A common approach for evaluating the likelihood function in Bayesian inference problems is to assume that the variance-covariance matrix of the error associated with the experimental observations (i.e., Σ) is a scaled identity matrix (i.e., the matrix is diagonal with all non-zero elements identical). However, the error models associated with an observation may depend highly on the experimental assay selected [29]. In addition, the data used in calibrating the model may correspond to different experimental studies and different techniques. Given that Σ is unknown *a priori *in most practical problems, Box and Draper noted that the marginal conditional probability, *P*(*Y*|Θ, *M*), can be obtained by assuming a prior for *P*(Σ) equal to |Σ|^-(*k*+1)/2 ^[33]. The corresponding likelihood relationship, of the form of a Wishart distribution, can be reduced analytically to a simple form expressed in terms of the determinant of the dispersion matrix:(5)

Similarly, an approximate Bayesian approach was recently described that uses a likelihood function equal to the determinant of the dispersion matrix alone [16]. In practice, the dispersion matrix can be normalized to a reference quantify to minimize the contribution of round-off error in calculating *P*(*Y*|Θ, *M*). Given the rugged landscape of the dispersion matrix with respect to the parameter space, a closed form solution of the expectation integral (i.e., Equation 1) is intractable. As an alternative, Monte Carlo sampling is an efficient method for integrating such complex integrals [32]. In the following section, a Monte Carlo algorithm is proposed that provides a Bayesian estimate of *P*(Θ|*M*, *Y*).

A Bayesian estimate for *P*(Θ|*M*, *Y*) can be obtained using computer-intensive methods. The Metropolis-Hasting (MH) algorithm [34,35] is a Monte Carlo technique that creates a sequence (i.e., a chain) of *θ*s that, in the limit of a long chain are drawn from the target distribution, *P*(Θ|*M*, *Y*). When the proposed steps are drawn from a stationary distribution, the resulting chain is a Markov chain. A proposed new step in the Markov chain, *θ*_*N*_, is accepted based upon the relative conditional probability of the new step in capturing the observations (i.e., *P*(*Y*|*θ*_*N*_, *M*)) relative to the current step. This acceptance criteria enables the collective Markov chain to represent samples drawn from the target distribution *P*(Θ|*M*, *Y*). New proposed steps within the Markov chain are drawn from a proposal distribution ∏(·). As mentioned above, the challenge in implementing a Markov Chain Monte Carlo (MCMC) approach is specifying the proposal distribution, ∏(·). In this study, the initial estimate of scale of proposal distribution for Θ was proportional to 1/*P*, where *P *is the row dimension of the vector Θ [36]. Parameter sampling was performed in log space to ensure that all kinetic parameters and species concentrations remain positive. In high dimensional problems with expensive calculations of the likelihood function, efficient sampling of *P*(Θ|*M*, *Y*) is essential and can be obtained using a proposal distribution that reflects the structure of the target distribution (i.e., the covariance of *P*(Θ|*M*, *Y*)).

A prior distribution, *P*(Θ), is used to provide an initial estimate of the proposal distribution, ∏(Θ). In conventional MCMC algorithms, the proposal distribution can be scaled to achieve a target acceptance fraction but the structure of the proposal distribution remains fixed to that specified by the prior distribution. A recent development in the MCMC field has been the development of adaptive MCMC (AMCMC) algorithms (e.g., [17]) that dynamically adjust the structure of the proposal distribution based upon the prior steps of an evolving Markov chain. The prior distribution used in this study was non-informative (i.e., the same for all parameters), proper (i.e., a finite integral), normally distributed (i.e., *N*(0, 1/*P*)), and used to specify the initial proposal distribution (∏(·)). Non-informative in this context means that no information was used to specify that there was greater uncertainty in some of the parameters relative to others. Following a specified "learning" period, the proposal distribution was adjusted, as described in the next section, to reflect the structure in the cumulative Markov chain.

#### Adaptive MCMC algorithm

The following adaptive MH algorithm was used to generate a Monte Carlo Markov Chain from multi-response data:

1. Start the chain using initial values for *θ*_0 _obtained via simulated annealing and an initial proposal distribution with the corresponding elements:(6)

where *P *is the row dimension of Θ, *δ*_*jk *_is the Kronecker's delta, and *s *is an adjustable proposal scaling factor used to give an initial acceptance fraction of 0.2. Select a value for *s*. Note that the Kronecker's delta, *δ*_*jk*_, is equal to 1 when the indices *j *and *k *are equal and *δ*_*jk *_is equal to 0 when the indices are not equal.

2. Using the current *θ*_*i *_of the Markov chain, propose a candidate step, *θ*_*N*_, that is drawn from ∏(·) according to(7)

which cumulatively represent a random walk through parameter space with the step size in a particular parameter direction determined by ∏(·).

3. Evaluate *P*(*Y*|*M*, *θ*_*N*_) using the form of the likelihood:(8)

where *Y*_*j *_is a vector of observed responses for experiment *j*, *M*_*j*_(*θ*_*N*_) is the corresponding vector of predicted responses using a model and the corresponding parameter values *θ*_*N*_, and *N*_*obs*, *j *_is the number of responses observed in experiment *j*.

4. Calculate the Metropolis acceptance probability:(9)

where *q*(·) is the jumping probability in the direction given as the argument. Given that, in this case, the proposal distribution is symmetric, the *q*(·) terms cancel.

5. Accept move *θ*_*i*+1 _= *θ*_*N *_with probability *h*, else retain current location *θ*_*i*+1 _= *θ*_*i*_.

6. Update *Cov*(*θ*_*i*+1_... *θ*_0_) and *s *every *x *AMCMC steps.

7. Using the current values for *Cov*(Θ) and *s*, update the proposal distribution whereby elements of ∏(·) are calculated using the following adaptive relationship:(10)

where *Chol*_*jk*_(·) is the *jk *element of the upper triangular matrix obtained from Cholesky factorization of the covariance matrix, *Cov*(·), and *N*_*L *_is the number of steps contained in an initial non-adapting "learning" period. As proposal density is symmetric, the condition of a detailed balance among random steps within parameter space is satisfied using *h *as an acceptance probability.

8. Go to 2.

The algorithm was implemented in Matlab (Natick, MA), numerical integration of the set of differential algebraic equations was obtained using the SUNDIALS IDA solver (Lawrence Livermore National Laboratory, Livermore, CA), and R/Bioconductor was used for the analysis of the Markov chain. In addition, the Markov Chains generated by this AMCMC algorithm converge to a stationary target distribution for *P*(Θ|**Y**, *M*) (i.e., is ergodic) as the adaptation of the proposal distribution exhibits diminishing adaptation (i.e., lim_*i*→∞_||∏_*i *_- ∏_*i*-1_|| = 0) [37].

An inherent characteristic of a Markov chain generated using a Metropolis-Hasting algorithm is the autocorrelative structure of the chain. The presence of autocorrelation between subsequent steps biases estimating the covariance of the model parameters. Mixing is a term used to characterize the autocorrelative structure of the chain. In well-mixed chains, steps separated by a specified distance within the chain exhibit minimal autocorrelation. To minimize the effect of autocorrelation, independent samples from the posterior distribution can be obtained by selecting values from the Markov Chain at every *n*th iteration, a technique called "thinning." Thinning was used to improve recursive calculation of the proposal covariance of the Markov Chain during the MCMC run. A thinning value of 40 was used to estimate the covariance recursively from the evolving Markov chain, while a thinning value of 200 was used to obtain *P*(Θ|**Y**, *M*) from the final Markov chains.

#### Convergence of Markov chain

One of the challenges with implementing a MCMC approach for Bayesian inference is deciding when the cumulative Markov chain is a representative sample drawn from the underlying stationary distribution. A Markov chain represents a random walk within parameter space weighted by the relative conditional probability of each step. Convergence is a criteria used to evaluate how long of a chain is necessary to traverse a representative sample of parameter space. Numerous algorithms have been developed to diagnose the convergence of a Markov chain [38]. Most conventional applications of these convergence criteria have focused on the model parameters. However, models of cellular signal transduction pose significant challenges for model-based inference and require a non-conventional approach.

As the objective of this study is to make statements of belief about the predictions of the model rather than particular values of the parameters, a convergence criterion was developed based upon the predictions of the model. The focus on predictions rather than the parameters was motivated by timescale considerations. When a complex dynamical system experiences an abrupt change in environmental conditions, the response of the system can be simplified into different kinetic manifolds (e.g., [39]). This phenomenon has been termed the slaving principle [40]. The evolution in the system is constrained by the slow variables (i.e., the slow kinetic manifold) while the fast variables (i.e., the fast kinetic manifold) exist at a pseudo-equilibrium. An implication of the slaving principle on Bayesian inference is that the posterior distributions of parameters may exhibit one-sided distributions. The parameters associated with a variable contained within the fast kinetic manifold may be increased above a threshold value with minimal impact on the dynamic response of the system. Below the threshold value, the dynamics of the corresponding variables impinge upon the slow kinetic manifold altering the dynamic response of the system and fitness of the model. In practice, the upper bounds of the parameters are defined by the tolerance of the ODE solver to solve systems with divergent timescales. To surmount this computational challenge, the Gelman-Rubin method was used for assessing convergence of model predictions derived from a series of three parallel Markov chains [41,42]. The method is based upon the concept that convergence has been achieved when the variance among chains is less than within single chains.

To estimate convergence, we use the prediction, *PY*_*ij*_, obtained from a single draw from *J *parallel MCMC samples of length *N*, where *j *∈ *J *and *i *∈ *N*. To illustrate the calculations, a simplified notation is used where *PY*_*ij *_represents the state of species *k *at time *m *(i.e., (*t*_*m*_, *θ*_*ij*_)). For each prediction, the between-sequence (*B*) and within-sequence (*W*) variances for *PY*_*ij *_are computed as follows:(11)

where  is the mean of the *N *predictions obtained from the *j *-*th *sequence:(13)

 is the mean of the predictions across sequences:(14)

and the sample variance of the predictions for the *j - th *sequence, *S*_*j *_is given by:(15)

The overall variance of the predictions derived from the target distribution is estimated from *W *and *B *by:(16)

The Gelman-Rubin method diagnoses convergence from the potential scale reduction factor:(17)

whereby parallel chains of length *N *should be increased until  is less than 1.2 [42]. These converged parallel chains represent samples drawn from a stationary distribution. In conventional use, the Gelman-Rubin method is applied to the second *N *iterations drawn from parallel chains, each of length 2*N*. The first *N *steps are discarded as they are assumed to be drawn from tails of the stationary distributions. Due to the cost of these calculations, only the "learning" period was discarded. The remainder of the parallel chains were used to estimate the convergence of the predictions to a stationary distribution.

## Results

### Model calibration

The mathematical model, shown schematically in Figure 1, was calibrated against values obtained from the literature. The values reported in the literature were obtained largely in primary rat hepatocytes in response to 20 nM EGF. Kinetic parameters associated with EGF binding to ErbB1 and affinities for protein-protein interactions were used from the literature to reduce the number of free parameters. Dissociation constants (*K*_*D*_'s) for particular protein-protein interactions, where reported, were also obtained from the literature [27,28] (see Additional file 1: Table S5). In addition, dissociation constants for reactions that create a cyclic pathway (e.g., reaction classes 8, 11, 14, and 15) were constrained by thermodynamic considerations. In addition, the initial values for Grb2, PLC-*γ*, Shc, Sos, RasGTP, and RasGDP were determined using simulated annealing. A list of all of the parameters can be found in (see Additional file 1: Table S3). The initial concentration of PTP was specified to be 100 nM, as the initial concentration of PTP was confounded with other parameters (data not shown). The initial values for protein expression are dependent on the assumed dissociation constants for protein-protein interaction. Similar dynamics of the system are observed if systematic reductions in the *K*_*D*_'s of protein-protein interaction are coupled with corresponding decreases in the concentration of the signaling proteins (data not shown). Given the best fit values determined using simulated annealing, an empirical Bayesian approach was used to estimate the uncertainty in the model parameters given the available calibration data.

### An empirical Bayesian approach to sensitivity analysis

**Figure 2:**
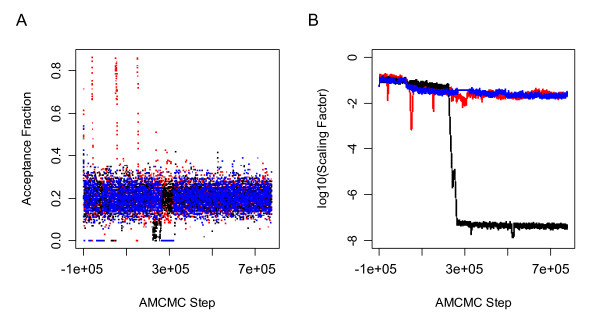
**Evolution in the proposal covariance scaling factor and the acceptance fraction as a function of AMCMC step**. The proposal density is scaled dynamically to achieve an acceptance fraction of 0.2. (A) The trace of the acceptance fraction is shown as a function of AMCMC step. (B) The trace of the covariance scaling factor is shown as a function of AMCMC step. The results for each of the three parallel chains are shown in different colors: Chain 1 (Blue), Chain 2 (Black), and Chain 3 (Red).

**Figure 3:**
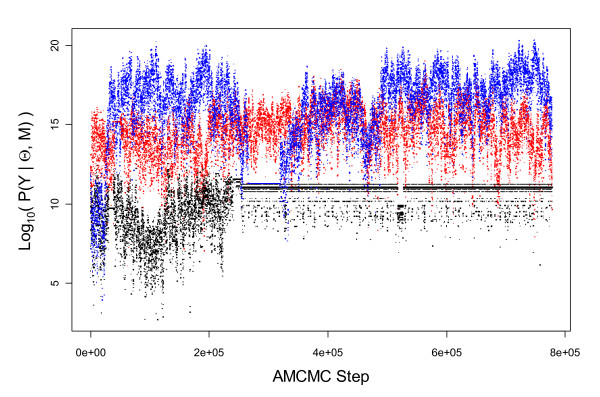
**Evolution in the likelihood, *P*(*Y *|Θ, *M*), as a function of AMCMC step**. The normalized likelihood value shown as a function of AMCMC step for all three parallel chain. The results for each of the three parallel chains are shown in different colors: Chain 1 (Blue), Chain 2 (Black), and Chain 3 (Red).

**Figure 4:**
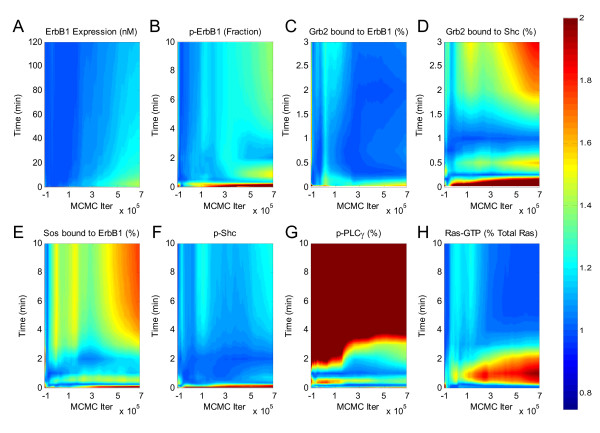
**Convergence of AMCMC algorithm**. A contour plot of the Gelman-Rubin statistic (i.e., the z-axis equals the potential scale reduction factor -) of the model predictions as a function of time (i.e., the y-axis) calculated as a function of the cumulative chain up to a specific AMCMC step (i.e., the x-axis). Three parallel chains were used to calculate the Gelman-Rubin statistics for the simulated (A) total ErbB1 expression, (B) phosphorylated form of ErbB1, (C) total cellular Grb2 bound to activated ErbB1, (D) percentage of total Grb2 bound to activated Shc, (E) percentage of total Sos bound to activated ErbB1, (F) phosphorylated form of Shc, (G) phosphorylated PLC*γ*-1, and (H) active Ras (RasGTP). Values less than 1.2 suggest convergence of the chains.

A series of Markov chains generated using a Metropolis-Hasting algorithm were used to estimate the conditional uncertainty in the model parameters. Three parallel Markov chains were calculated each containing 900,000 steps. The simulation of each chain took approximately 550 hours on a single core of a 2.66 GHz Dual-Core Intel Xeon 64-bit processor with 8 GB RAM. The parameter values obtained using simulated annealing provided the starting point for the chain. A "learning" (a.k.a. "burn-in") period of 100,000 steps was specified *a priori *to provide an initial estimate for the proposal covariance. In addition, this "learning" period was used to correct for starting values of the chain that may correspond to samples from the tail of *P*(Θ|**Y**, *M*). Following the "learning" period, ∏(·) was adjusted to correspond to the covariance of the cumulative target distribution *P*(Θ|**Y**, *M*). The trace of the acceptance fraction (Panel A in Figure 2) demonstrate that the scaling factor was adjusted at regular intervals (see Panel B in Figure 2) to maintain the acceptance fraction around 0.2. The trace of the conditional probability for each of the three chains, shown in Figure 3, suggests that Markov chains were no longer sampling from the tails of the posterior distributions, as may occur during the "learning" period. One of the chains (see black chain in Figure 3) entered a stiff region of parameter space whereby numerical difficulties in selecting an appropriate integration step size retained the Markov chain within this region. The Gelman-Rubin potential scale reduction factor (PSRF) was applied to the model predictions to assess the convergence of the cumulative Markov Chains obtained following the "learning" period. The Gelman-Rubin PSRF statistics were calculated for the species observed experimentally as a function of time and AMCMC step and shown graphically as a contour plot in Figure 4. The colored contours correspond to values of the PSRF. Many of the model predictions exhibited a potential scale reduction factor of below 1.2 immediately after the "learning" period (e.g., ErbB1 expression, phosphorylated ErbB1, Grb2 bound to ErbB1, and phosphorylated Shc) while other predictions were unable to converge for the entire prediction window (e.g., phosphorylated PLC-*γ *at times greater than 2 minutes). Given the variability in convergence, the entire chains, following the initial learning period, were used in the final analysis of the Markov chains. The maximum expectation values determined using the AMCMC algorithm are also reported (see Additional file 1: Table S3). In addition, the PSRF applied to the parameters are also shown for comparison (see Additional file 1: Table S3).

**Figure 5:**
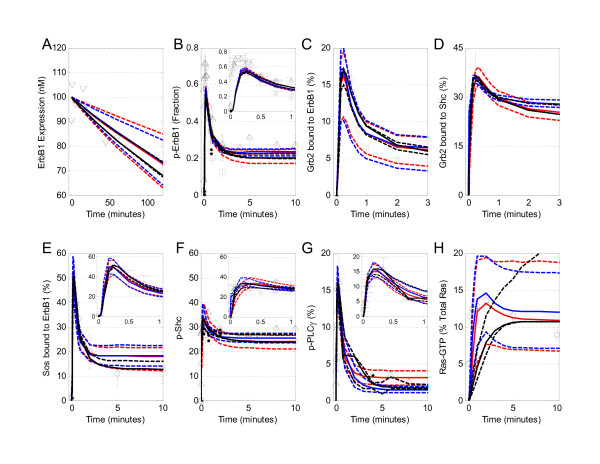
**Model calibration simulations**. The mathematical model for early signaling events reproduces the early dynamics for activation of selected pathways downstream from ErbB1. Simulated results (lines) are compared against the experimental observations (symbols) used to calibrate the mathematical model. The uncertainty in the model predictions obtained from each are represented by three lines of the same color: the most likely prediction is represented by the solid lines and the dashed lines represent the 95th and 5th percentile of the predicted response. The results for each of the three parallel chains are shown in different colors: Chain 1 (Blue), Chain 2 (Black), and Chain 3 (Red). (A) Simulated response (lines) versus measured total ErbB1 expression in 184A1 cells (▽[20]). (B) Comparison to the phosphorylated form of ErbB1 was reported as a percentage of the total ErbB1 expression (○ [18], □ [30], and △ [31]). (C) The simulated fraction of total cellular Grb2 bound to activated ErbB1 (line) is compared against experimental values (○ [18]). (D) Simulated (line) and measured (circles) [18]) percentage of total Grb2 bound to activated Shc. (E) Simulated (line) and measured (□ [30]) percentage of total Sos bound to activated ErbB1. (F) The phosphorylated form of Shc, reported as a percentage of total Shc (○ [18], △[31], ◇ [22]), is compared against the simulated value (line). (G) Comparison of simulated (line) versus the phosphorylated form of PLC*γ*-1, reported as a percentage of total PLC*γ*-1 (△ [31]). (H) The active form of Ras (RasGTP), reported as a percentage of total Ras (□ [30]), is compared against the simulated values (line). The initial conditions used in the simulation are tabulated (see Additional file 1: Table S3). With the exception of p-Shc observed by [22], all observations were made following exposure to 20 nM EGF in primary rat hepatocytes. In panel F, the measurements by [22] were obtained following exposure to 10 nM EGF in MCF-7 cells.

**Figure 6:**
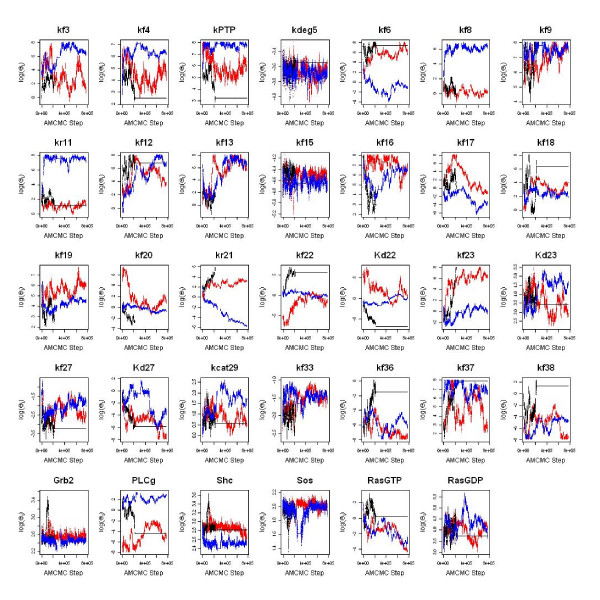
**AMCMC summary plots for each of the model parameters**. The trace of each of the model parameters is shown as a function of MCMC step. The traces for three parallel chains are shown in different colors: Chain 1 (Blue), Chain 2 (Black), and Chain 3 (Red).

A common feature seen in the different subplots of Figure 4 (e.g., subpanels B, D, and E) was that the variability among chains, as represented by an increase in PSFR, increased as a function of simulation time (i.e., the vertical axis). This behavior was due to undersampling at longer times relative to earlier times. Oversampling of a region implicitly applies a higher relative weight in estimating *P*(*Y*|*θ*_*N*_, *M*). For comparison, the 95th percentile, 5th percentile, and median responses for each of the three chains are overlaid upon the experimental data in Figure 5. Traces for each of the parameters are shown in Figure 6.

## Discussion

In this study, an empirical Bayesian approach was developed to quantify the uncertainty in the estimated parameters, given the available data, and to estimate the uncertainty in the model predictions, given the range of plausible parameter values. In contrast to recent efforts that use proposal distributions that have fixed structures (e.g., [11-15,43]), the empirical Bayesian approach used in this study was based upon an adaptive Markov chain Monte Carlo algorithm that adjusts the structure of the proposal distribution based upon the covariance of the cumulative Markov chain. In another recent publication, we used this algorithm to infer the relative strength of control mechanisms in the Interleukin-12 signaling pathway observed in naïve CD4+ T cells by our own hands (Finley SD, Gupta D, Cheng N, Klinke DJ: Inferring Relevant Control Mechanisms for Interleukin-12 Signaling in Naïve CD4+ T Cells, submitted). In this study, an AMCMC algorithm was used to help interpret measurements of Epidermal Growth Factor signaling observed in rat hepatocytes by others using signaling mechanisms postulated from the literature.

**Figure 7:**
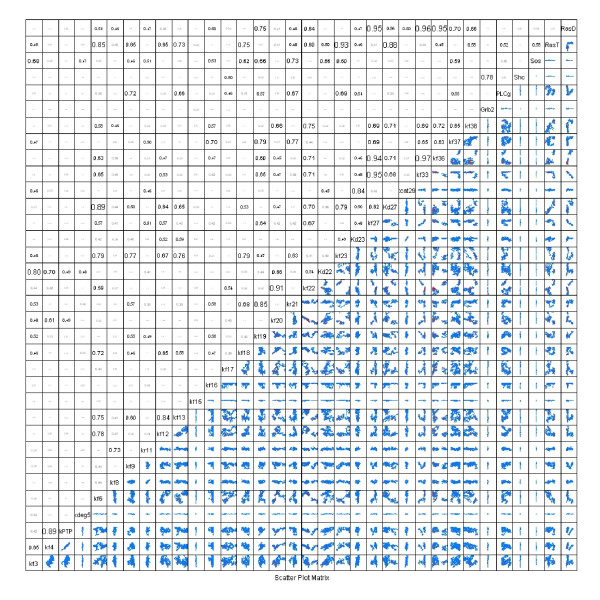
**Covariance structure of *P*(Θ| Y)**. The pairwise correlation coefficients of the parameters derived from all three thinned (*K*_*thin *_= 200) Markov chains are shown above the diagonal. A high value for the correlation coefficient suggests that the parameters are unidentifiable given the calibration data. Below the diagonal, pairwise projections of the marginalized probability density for *P*(Θ|**Y**) are shown. The parameter names are shown on the diagonal. Each scatter plot axis spans a range of 10^12 ^(i.e., (*log *- *mean *- 6.0 to *log *- *mean *+ 6.0). The values in each scatter plot are centered at the log-mean values (i.e., the expectation maximum) determined from three parallel Markov Chains each containing 800,000 MCMC steps.

Following an assessment of the convergence of the parallel Markov chains, pairwise scatter plots were used to illustrate the structure in the posterior distributions of the parameter values (see Figure 7). Pairwise comparisons of the expected parameter values showed that a subset of the parameter values were not independent but exhibited interdependence, as illustrated by diagonal structure in the scatter plots (see subplot for RasGDP versus *k*_*f*33 _in Figure 7). Pairwise correlation coefficients are shown above the diagonal. Analysis of the covariance of the thinned Markov chains, shown in Figure 7, suggests that parameters RasGDP and *k*_*f*33 _are not identifiable as they exhibit a negative correlation coefficient of 0.95. This was expected as increase in the rate of RasGTP hydrolysis (i.e., an increase in *k*_*f*33_) can compensate for low levels of RasGDP. The density plots, shown to the right of the diagonal in Figure 7, suggest that the initial concentrations of Grb2, Shc, and Sos are tightly constrained given the available data. In addition, *k*_*deg*5 _and *k*_*f*15 _were two of the kinetic parameters that were tightly constrained. In contrast, many of the parameters (e.g., *k*_*f*3 _and *k*_*f*17_) exhibited a much larger uncertainty range (i.e., greater than O(10^10^)). It is also interesting to note that - even though Shc, Sos, and Grb2 were constrained by the data - the initial concentrations of PLC*γ *and RasGTP/RasGDP were not tightly constrained. This difference can be attributed to the calibration data for PLC*γ *and RasGTP/RasGDP were reported in terms of relative amounts instead of absolute quantities and these species were specified as terminal species in the model. The broad range in posterior distribution of PLC*γ *expression reflects the inability of the Markov chain to converge in predicting the phosphorylation state of PLC*γ *at longer times. Future simulations that include an estimation of initial concentration of PLC*γ *within this cell type may aid in convergence.

Estimated values of the PSRF applied to the parameters (see Additional file 1: Table S3) are consistent with the pairwise scatter plots where parameters with low variance converge first (e.g., *k*_*f*15 _and *k*_*deg*5_) while parameters with high variance require a long chain to converge (e.g., *k*_*f*6_, *k*_*f*23_, and *k*_*f*38_). In general, the model results were sensitive to the initial concentrations of Grb2, Shc, and Sos as the posterior distributions were narrower than many of the other parameters shown in Figure 7. A similar observation was reported by Kholodenko et al. [18] and highlights how variations in protein expression influence cellular response. The rate of degradation of phosphorylated ErbB1 (*k*_*deg*5_) and the rate of PTP binding to phosphorylated ErbB1 (*k*_*f*15_) exhibited the narrowest distributions of all of the parameters. Despite the uncertainty in the parameter values, the model predictions are confined within a relatively narrow region (see Figure 5). By marginalizing the predicted responses over *P*(Θ|**Y**), this range in model predictions provided an estimate of *P*(|*M*).

### Efficiency of sampling parameter space

High dimensional problems are particularly challenging for MCMC algorithms due to the large volume of parameter space that must be searched. The existence of correlation among the parameters and different timescales inherent in the problem compound this challenge. The contours of the proposal density distribution of Θ enclose an *P*-dimensional hyperellipsoid. The volume of this ellipsoid (*Vol*_*P*_) is proportional to the determinant of the proposal density by:(18)

**Figure 8:**
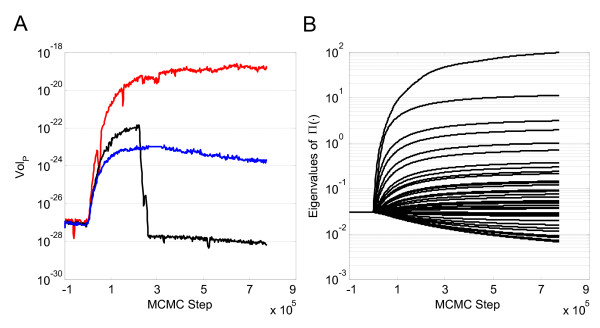
**Evolution in the proposal density distribution as a function of AMCMC step**. (A) A value proportional to the volume of the *P*-dimensional hyperellipsoid that encloses the proposal density distribution (∏(·)) for the AMCMC algorithm shown as a function of MCMC step for Chain 1. (B) The change in the eigenvalues of ∏(·) as a function of MCMC step indicate the contraction and expansion along different orthogonal directions of the hyperellipsoid, shown for one of the parallel chains.

A plot of the right hand side of Equation 18 shown as a function of AMCMC step is shown in Figure 8A. Following the "learning" period, the proposal distribution rapidly expanded to reflect the structure of the cumulative Markov chain and established a stationary proposal distribution after an additional 200,000 steps. The structure of the proposal distribution was slightly different for each of the three parallel chains, as shown in Figure 8A. In the AMCMC algorithm, ∏(·) is obtained from the covariance of *P*(Θ|*M, Y*). Conceptually, an eigenvector/eigenvalue decomposition of the covariance of *P*(Θ|*M, Y*) provides a convenient framework to rationalize about the structure of the parameter space. In a well-posed problem, the unit orthogonal vectors (i.e., eigenvectors) of the parameter space exhibit finite length (i.e., non-zero eigenvalues). High correlation between model parameters effectively reduce the dimension of the parameter space and reduce the length of one or more of the orthogonal vectors to zero. This dimensional reduction implies that the hyperellipsoid lies wholly in a (*P *- 1^+^)-dimensional subspace and the volume in *P*-space is zero. The efficiency of the AMCMC algorithm is that it samples from the (*P *- 1^+^)-dimensional subspace rather than *P*-space. The expansion and contraction of parameter space in the different orthogonal parameter directions, as shown by the change in eigenvalues as a function of AMCMC step, is shown in Figure 8B. As specified in the AMCMC algorithm, the unit dimensions are constant during the "learning" period and adapt following this initial period.

### Model-based inference of cell signaling pathways

Rationalizing about the biological mechanisms that underpin biological response can be aided using mathematical models. A mathematical model allows for the prediction of a series of response variables, , given a model topology, *M*, and a set of parameter values, Θ, determined from some calibration data, *Y*. Biological responses can be explained using mathematical models with finite accuracy. This accuracy is in part limited by uncertainty associated with measured data. The uncertainty in experimental measurement can be attributed to technical variation (e.g., intrinsic error associated with a particular assay) or biological variation (e.g., cellular heterogeneity, asynchrony, or stochasticity). The variance may vary between experimental assays of the same biological system and limit observing significant trends in the response variables (e.g., [29]). Mathematically, the relationship between experimental observations, mathematical predictions, and uncertainty can be related by:(19)

where *δ*(*Y*) are systematic differences between the model and data and ϵ is a random error component that reflects the inherent uncertainty of a particular assay to probe biology. In analyzing measured data using mathematical models, it is typically assumed that ϵ is independently and identically distributed with a mean of zero and *δ*(*Y*) is negligible. These assumptions provide the basis for likelihood functions, such as Equation 3, and some cost functions, such as root mean squared error, used in optimization algorithms. Optimization focuses on finding the best solution to a problem given a set of constraints, such as finding *P*(|*M, Y*) such that ϵ is a minimum. Optimization and other aspect related to fitting a model to data was recently reviewed with respect to systems biology [44]. However, there are two aspects of analyzing data using mathematical models that deserve further clarification with respect to the empirical Bayesian approach described herein.

The first aspect is related to the model predictions, *P*(|*M*, *Y*). A single point estimate for *P*(|*M, Y*) (i.e., the maximum likelihood estimate: *MLE*(|*M, Y*)) is the most commonly used approach to generate model predictions [7]. However, the model predictions are also dependent on the particular parameter values used in the prediction. A better estimate of model adequacy can be obtained by reporting the uncertainty associated with the predictions of the model given a range in plausible parameter values (i.e., the posterior distribution of the parameters). The uncertainty in the predictions can be obtained by integrating over the posterior distribution in the parameter values:(20)

In practice, marginalizing the predicted response variables over the converged Markov chain provided a numerical integration of Equation 20.

The second aspect is the uncertainty associated with what the model is unable to predict:(21)

**Figure 9:**
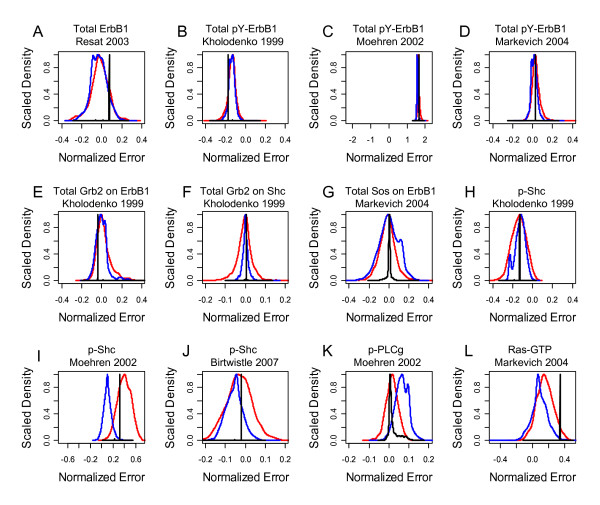
**Normalized error of the model predictions stratified by study**. The distribution in the sum of the normalized error between the calibration data and the model predictions is shown for each study used in the analysis. (A) Total ErbB1 expression [20], (B) total phosphorylated ErbB1 [18], (C) total phosphorylated ErbB1 [31], (D) total phosphorylated ErbB1 [30], (E) total Grb2 attached to ErbB1 [18], (F) total Grb2 attached to Shc [18], (G) total Sos attached to ErbB1 [30], (H) phosphorylated Shc [18], (I) phosphorylated Shc [31], (J) phosphorylated Shc [22], (K) phosphorylated PLC-*γ *[31], and (L) activated Ras (Ras-GTP) [30]. Distributions for the three chains are shown in different colors: Chain 1 (Blue), Chain 2 (Black), and Chain 3 (Red).

The absence of systematic trends instills confidence in both the model predictions and the experimental data. Variance that exhibits systematic trends (i.e., *δ*(*Y*)) implies that features of the biological system are not represented in the mathematical model or that inconsistencies exist within the experimental data. When systematic trends are observed, it may be tempting to discard the model as inadequate. However, a closer examination of *δ*(*Y*) may provide insight into aspects of the underlying biology as trends identify gaps in knowledge. These gaps in knowledge are also opportunities for discovery. For the EGF model analyzed using the empirical Bayesian approach, the normalized error, shown in Figure 9, provided an estimate of *δ*(*Y*) for each measurement used in calibrating the model. The distributions in *δ*(*Y*) for Total ErbB1, Total Grb2 on ErbB1, Total Grb2 on Shc, and Total Sos on ErbB1 were distributed around zero, suggesting the absence of systematic trends. However, a common approach in the field is to collect calibration data from a variety of sources and systems due to the lack of data on a specific system. In that case, systematic trends may reflect inherent variation in response among systems. A more detailed analysis of the calibration data identified inconsistencies within the experimental data. The comparison among the different reported measurements of Total pY-ErbB1 was interesting. The model predictions largely capture the values reported by Markevich et al. (i.e., *δ*(*Y*) is centered around zero), consistently underpredict the values reported by Moehren et al. (i.e., *δ*(*Y*) is greater than zero), and consistently overpredict the values reported by Kholodenko et al. The earlier time points for ErbB1 phosphorylation was reported by Moehren et al. compared to the other studies while the data reported by Kholodenko appears to exhibit a more rapid decline than observed by Markevich et al. (see panel B in Figure 5). It may seem that an increase in the EGF binding rate may provide better agreement with the Moehren et al. data. However, the rate constant for EGF binding was obtained from the literature [45] and is a factor of 10^5 ^slower than the other steps that lead to ErbB1 phosphorylation. Additional studies may be needed to rectify these seemingly conflicting observations.

Differences between the expected behaviors and new data identify areas where our understanding of the system is inadequate and reveal novel aspects of biology [6]. In the case of signal transduction models, systematic differences may be used to discriminate between competing mechanistic descriptions, such as between recent bench experiments that report aspects of Ras activation [26] or of dose-dependent aspects of EGF stimulation [46] and those represented in current models [47]. While developing an empirical Bayesian approach was the primary focus of this work, two additional points deserve mention in analyzing the EGF signaling data. First, the lack of convergence for the Ras predictions suggests that additional information, such as a more quantitative estimate for Ras expression levels within rat primary hepatocytes, are required before a level of belief can be stated regarding the agreement between the new mechanistic aspect of Ras activation and the observed dynamics. Second, an underlying assumption is this model is that all of the reactants are in the same compartment (i.e., localized nearby the plasma membrane). However, a common theme for a variety of signaling receptors suggests that cellular response to receptor stimulation is dependent on the receptor's subcellular location (e.g., [48-50]). Regarding EGF signaling, Vieira et al. reported that PLC*γ *exhibited an increase in phosphorylation in cells with defective clathrin-mediated endocytosis [51]. This underlying biology may contribute to the inability for the PLC*γ *predictions to converge. Similar to the Ras predictions, quantitative estimates for PLC*γ *expression levels within rat primary hepatocytes may help improve the model predictions for PLC*γ*. Finally, this work helps set the framework for coupling empirical Bayesian techniques with information theory, such as the Akaike Information Criterion [52-54] or Bayes Factor (e.g., [12,16]), to establish a level of confidence with distinguishing between competing mechanisms of information flow.

A recent study by Chen and coworkers elevates the standard for developing and testing mathematical models of cell signaling networks [47]. In this paper, the uncertainty associated a mathematical model of the canonical pathways associated with the EGF family of receptors, ErbB1 - ErbB4, was estimated using a ensemble of parameter point estimates obtained by simulated annealing. While stochastic sampling of parameter space is an essential requirement for an algorithm to navigate successfully within a rugged landscape with multiple local minima, using a model to make inferences about cell signaling networks requires that the observed samples are representative samples derived from the posterior distribution.

While the dimensionality of the model described by Chen et al. may be too computationally expensive for a conventional Bayesian approach, AMCMC techniques, as described here, provide an attractive approach to sample high-dimensional parameter space efficiently. Moreover, demonstrating that Markov chain is a representative sample of the posterior distribution with a convergence criterion applied to the parameters can make a conventional Bayesian approach computationally intractable due to the presence of different timescales. As described here, the convergence criterion can be applied to the model predictions thereby simplifying the computational burden of the approach.

## Conclusion

A common challenge in systems biology is to infer mechanistic descriptions of biological process given limited observations of a biological system [55,56]. In the area of signal transduction, mathematical models are frequently used to represent a belief about the causal relationships among proteins within a signaling network. The simulated dynamics of these models are dependent on the topology of the signaling network and the parameters associated with each protein-protein interaction. An outstanding question is whether a specific topology can capture the observed biology given a range of plausible parameter values. By integrating over the range of plausible parameter values, we may establish a level of confidence with a specific topology. If the level of confidence is low, we may learn that we have left out a critical link within the signaling network. A Bayesian perspective may be particularly helpful for determining aspects of the biology that are inconsistent with the stated hypothesis about how information flows within a network. As illustrated by the example developed in this paper, models of signal transduction frequently synthesize information derived from a variety of experimental studies. Tools, such as the AMCMC algorithm and Gelman-Rubin convergence metrics, that facilitate evaluating different sources for this information may also be helpful. In summary, an empirical Bayesian approach was developed for inferring the confidence in a particular model for describing signal transduction mechanisms and for inferring inconsistencies in experimental measurements.

## Supplementary Material

Additional file 1 **Model Definition**. A series of tables that define the mathematical model, parameter values, and initial conditions used in this manuscript. Click here for file

## References

[B1] Asthagiri AR Lauffenburger DA Bioengineering Models of Cell Signaling Ann Rev Biomed Eng 2000 2 31 53 10.1146/annurev.bioeng.2.1.31 11701506

[B2] Lazebnik Y Can a biologist fix a radio?--Or, what I learned while studying apoptosis Cancer Cell 2002 2 179 183 10.1016/S1535-6108(02)00133-2 12242150

[B3] Anderson AR Quaranta V Integrative mathematical oncology Nat Rev Cancer 2008 8 3 227 234 10.1038/nrc2329 18273038

[B4] Yaffe MB Signaling Networks and Mathematics Sci Signal 2008 1 eg7 10.1126/scisignal.143eg7 18957689

[B5] Broadbelt LJ Pfaendtner J Lexicography of kinetic modeling of complex reaction networks AIChE J 2005 51 2112 2121 10.1002/aic.10599

[B6] National Research Council (US) Committee on Learning, Research, Practice and Education How people learn: brain, mind, experience, and school 2000 Washington, DC: National Academies Press

[B7] Banga JR Optimization in computational systems biology BMC Sys Bio 2008 2 47 10.1186/1752-0509-2-47 PMC2435524 18507829

[B8] Gelman A Carlin JB Stern HS Rubin DB Bayesian Data Analysis 2004 Texts in Statistical Science, Boca Raton, FL: Chapman and Hall

[B9] Gamerman D Markov Chain Monte Carlo Stochastic simulation for Bayesian inference 1997 New York, NY: Chapman & Hall USA

[B10] Wilkinson DJ Bayesian methods in bioinformatics and computational systems biology Briefings in Bioinformatics 2007 8 2 109 116 10.1093/bib/bbm007 17430978

[B11] Coleman MC Block DE Bayesian parameter estimation with informative priors for nonlinear systems AIChE J 2006 52 2 651 667 10.1002/aic.10667

[B12] Vyshemirsky V Girolami MA Bayesian ranking of biochemical system models Bioinformatics 2008 24 6 833 839 10.1093/bioinformatics/btm607 18057018

[B13] Rogers S Khanin R Girolami M Bayesian model-based inference of transcription factor activity BMC Bioinformatics 2007 8 Suppl 2 S2 17493251 10.1186/1471-2105-8-S2-S2 PMC1892071

[B14] Battogtokh D Asch DK Case ME Arnold J Schuttler HB An ensemble method for identifying regulatory circuits with special reference to the qa gene cluster of Neurospora crassa Proc Natl Acad Sci USA 2002 99 26 16904 16909 12477937 10.1073/pnas.262658899 PMC139242

[B15] Brown KS Sethna JP Statistical mechanical approaches to models with many poorly known parameters Phys Rev E 2003 68 2 021904 10.1103/PhysRevE.68.021904 14525003

[B16] Toni T Welch D Strelkowa N Ipsen A Stumpf MPH Approximate Bayesian computation scheme for parameter inference and model selection in dynamical systems Journal of the Royal Society Interface 2009 6 31 187 202 10.1098/rsif.2008.0172 PMC2658655 19205079

[B17] Haario H Saksman E Tamminen J An Adaptive Metropolis Algorithm Bernoulli 2001 7 223 242 10.2307/3318737

[B18] Kholodenko BN Demin OV Moehren G Hoek JB Quantification of Short Term Signaling by the Epidermal Growth Factor Receptor J Biol Chem 1999 274 30169 30181 10.1074/jbc.274.42.30169 10514507

[B19] Schoeberl B Eichler-Jonsson C Gilles ED Muller G Computational modeling of the dynamics of the MAP kinase cascade activated by surface and internalized EGF receptors Nat Biotechnol 2002 20 4 370 375 10.1038/nbt0402-370 11923843

[B20] Resat H Ewald JA Dixon DA Wiley HS An Integrated Model of Epidermal Growth Factor Receptor Trafficking and Signal Transduction Biophys J 2003 85 730 743 12885624 10.1016/S0006-3495(03)74516-0 PMC1303198

[B21] Blinov ML Faeder JR Goldstein B Hlavacek WS A Network Model of Early Events in Epidermal Growth Factor Receptor Signaling That Accounts for Combinatorial Complexity Biosystems 2006 83 136 151 10.1016/j.biosystems.2005.06.014 16233948

[B22] Birtwistle MR Hatakeyama M Yumoto N Ogunnaike BA Hoek JB Kholodenko BN Ligand-dependent responses of the ErbB signaling network: experimental and modeling analyses Mol Sys Bio 2007 3 144 http://www.biomedcentral.com/pubmed/18004277 10.1038/msb4100188 PMC2132449 18004277

[B23] Pawson T Nash P Assembly of Cell Regulatory Systems Through Protein Interaction Domains Science 2003 300 445 452 10.1126/science.1083653 12702867

[B24] Klinke DJ Broadbelt LJ Mechanism Reduction during Computer Generation of Compact Reaction Models AIChE J 1997 43 1828 1837 10.1002/aic.690430718

[B25] Klinke DJ Broadbelt LJ Construction of a Mechanistic Model of Fischer-Tropsch Synthesis on Ni(111) and Co(0001) Surfaces Chem Eng Sci 1999 54 3379 3389 10.1016/S0009-2509(98)00386-8

[B26] Gureasko J Galush WJ Boykevisch S Sondermann H Bar-Sagi D Groves JT Kuriyan J Membrane-dependent signal integration by the Ras activator Son of sevenless Nature Struct Mol Bio 2008 15 5 452 461 10.1038/nsmb.1418 PMC2440660 18454158

[B27] Jones RB Gordus A Krall JA MacBeath G A quantitative protein interaction network for the ErbB receptors using protein microarrays Nature 2006 439 168 174 10.1038/nature04177 16273093

[B28] Kaushansky A Gordus A Chang B Rush J MacBeath G A quantitative study of the recruitment potential of all intracellular tyrosine residues on EGFR, FGFR1 and IGF1R Molecular Biosystems 2008 4 6 643 653 10.1039/b801018h 18493663 PMC2811368

[B29] Klinke DJ Ustyugova IV Brundage K Barnett JB Modulating Temporal Control of NF-kappaB Activation: Implications for Therapeutic and Assay Selection Biophys J 2008 94 11 4249 4259 18281385 10.1529/biophysj.107.120451 PMC2480691

[B30] Markevich NI Moehren G Demin OV Kiyatkin A Hoek JB Kholodenko BN Signal processing at the Ras circuit: what shapes Ras activation patterns? Syst Biol 2004 1 104 113 10.1049/sb:20045003 17052120

[B31] Moehren G Markevich N Demin O Kiyatkin A Goryanin I Hoek JB Kholodenko BN Temperature dependence of the epidermal growth factor receptor signaling network can be accounted for by a kinetic model Biochemistry 2002 41 306 320 10.1021/bi011506c 11772030

[B32] Beers KJ Numerical Methods for Chemical Engineering. Applications in Matlab 2007 Cambridge: Cambridge University Press

[B33] Box GEP Draper NR The Bayesian Estimation of Common Parameters from Several Responses Biometrika 1965 52 355 365 10.1093/biomet/52.3-4.355

[B34] Metropolis N Rosenbluth AW Rosenbluth MN Teller AH Teller E Equation of state calculations by fast computing machine J Chem Phys 1953 21 1087 1091 10.1063/1.1699114

[B35] Hastings WK Monte Carlo sampling methods using Markov chains and their applications Biometrika 1970 57 97 109 10.1093/biomet/57.1.97

[B36] Gelman A Roberts GO Gilks WR Bernardo BJODAPJM, Smith AFM Efficient Metropolis Jumping Rules Bayesian Statistics 5 1996 Oxford University Press 599 607

[B37] Roberts GO Rosenthal JS Coupling and ergodicity of adaptive Markov chain Monte Carlo algorithms J Appl Prob 2007 44 2 458 475 10.1239/jap/1183667414

[B38] Cowles MK Carlin BP Markov chain Monte Carlo convergence diagnostics: A comparative review J Am Stat Assoc 1996 91 434 883 904 10.2307/2291683

[B39] Okino MS Mavrovouniotis ML Simplification of chemical reaction systems by time-scale analysis Chem Eng Comm 1999 176 115 131 10.1080/00986449908912149

[B40] Haken H Synergetics Introduction and Advanced Topics 2004 New York, NY: Springer-Verlag

[B41] Gelman A Rubin DB Inference from Iterative Simulation Using Multiple Sequences Stat Sci 1992 7 4 457 472 10.1214/ss/1177011136

[B42] Brooks SP Gelman A General methods for monitoring convergence of iterative simulations J Comp Graph Stat 1998 7 4 434 455 10.2307/1390675

[B43] Flaherty P Radhakrishnan ML Dinh T Rebres RA Roach TI Jordan MI Arkin AP A Dual Receptor Crosstalk Model of G-Protein-Coupled Signal Transduction PLoS Comp Bio 2008 4 9 e1000185 10.1371/journal.pcbi.1000185 PMC2528964 18818727

[B44] Jaqaman K Danuser G Linking data to models: data regression Nature Rev Mol Cell Bio 2006 7 813 819 10.1038/nrm2030 17006434

[B45] Wilkinson JC Stein RA Guyer CA Beechem JM Staros JV Real-time kinetics of ligand/cell surface receptor interactions in living cells: Binding of epidermal growth factor to the epidermal growth factor receptor Biochemistry 2001 40 34 10230 10242 10.1021/bi010705t 11513601

[B46] Macdonald JL Pike LJ Heterogeneity in EGF-binding affinities arises from negative cooperativity in an aggregating system Proc Natl Acad Sci USA 2008 105 112 117 18165319 10.1073/pnas.0707080105 PMC2224169

[B47] Chen WW Schoeberl B Jasper PJ Niepel M Nielsen UB Lauffenburger DA Sorger PK Input-output behavior of ErbB signaling pathways as revealed by a mass action model trained against dynamic data Mol Sys Bio 2009 5 239 http://www.biomedcentral.com/pubmed/19156131 10.1038/msb.2008.74 PMC2644173 19156131

[B48] Husebye H Halaas O Stenmark H Tunheim G Sandanger O Bogen B Brech A Latz E Espevik T Endocytic pathways regulate Toll-like receptor 4 signaling and link innate and adaptive immunity Embo Journal 2006 25 4 683 692 16467847 10.1038/sj.emboj.7600991 PMC1383569

[B49] Lei JT Martinez-Moczygemba M Separate endocytic pathways regulate IL-5 receptor internalization and signaling Journal of Leukocyte Biology 2008 84 2 499 509 18511572 10.1189/jlb.1207828 PMC2493073

[B50] Schutze S Tchikov V Schneider-Brachert W Regulation of TNFR1 and CD95 signalling by receptor compartmentalization Nature Reviews Molecular Cell Biology 2008 9 8 655 662 10.1038/nrm2430 18545270

[B51] Vieira AV Lamaze C Schmid SL Control of EGF receptor signaling by clathrin-mediated endocytosis Science 1996 274 5295 2086 2089 10.1126/science.274.5295.2086 8953040

[B52] Akaike H A New Look at the Statistical Model Identification IEEE Trans Automat Control 1974 19 716 723 10.1109/TAC.1974.1100705

[B53] Yamaoka K Nakagawa T Uno T Application of Akaike's information criterion (AIC) in the evaluation of linear pharmacokinetic equations J Pharmacokinet Biopharm 1978 6 165 175 10.1007/BF01117450 671222

[B54] Horn R Statistical methods for model discrimination. Applications to gating kinetics and permeation of the acetylcholine receptor channel Biophys J 1987 51 255 263 2435330 10.1016/S0006-3495(87)83331-3 PMC1329886

[B55] Gardner TS di Bernardo D Lorenz D Collins JJ Inferring Genetic Networks and Identifying Compound Mode of Action via Expression Profiling Science 2003 301 102 105 10.1126/science.1081900 12843395

[B56] Lorenz DR Cantor CR Collins JJ A network biology approach to aging in yeast Proc Natl Acad Sci USA 2009 106 1145 1150 19164565 10.1073/pnas.0812551106 PMC2629491

[B57] Le Novere N Moodie S Sorokin A Hucka M Schreiber F Demir E Mi H Matsuoka Y Wegner K Le Novere HKN Hucka M Mi H Moodie S Schreiber F Sorokin A Demir E Wegner K Aladjem MI Wimalaratne SM Bergman FT Gauges R Ghaza P Kawaji H Li L Matsuoka Y Villeger A Boyd SE Calzone L Courtot M Dogrusoz U Freeman TC Funahashi A Ghosh S Jouraku A Kim S Kolpakov F Luna A Sahle S Schmidt E Watterson S Wu G Goryanin I Kell DB Sander C Sauro H Snoep JL Kohn K Kitano H The Systems Biology Graphical Notation Nature Biotechnology 2009 27 8 735 741 10.1038/nbt.1558 19668183

