# Supplementary material for: An empirical Bayesian approach for model-based inference of cellular signaling networks
Source: BMC Bioinformatics. 2009 Nov 9;10:371. doi: 10.1186/1471-2105-10-371 (PMC2781012; doi:10.1186/1471-2105-10-371)
Supplement: Additional file 1 — Model Definition. A series of tables that define the mathematical model, parameter values, and initial conditions used in this manuscript. [file 1471-2105-10-371-S1.pdf]

Table S1 - List of model variables.

Variable Name	Definition	Units
E	Epidermal growth factor (EGF) ligand	nM
E_1	Unbound ErbB1 receptor	nM
EE_1	ErbB1 bound to EGF	nM
E_{11}	Dimer of EGF-bound ErbB1	nM
$E_{11}P$	EGF-bound ErbB1 dimers with phosphorylated tyrosines	nM
G	Unbound Grb2 in cytosol	nM
N	Unbound IRS-1 in cytosol	nM
PL	Unbound PLC-γ in cytosol	nM
Sh	Unbound Shc in cytosol	nM
ShP	Unbound phosphorylated Shc in cytosol	nM
S	Unbound Sos in cytosol	nM
T	Unbound Phosphotyrosine Phosphatase (PTP) in cytosol	nM
EYP	ErbB1 with unoccupied phosphorylated Tyr_x	nM
$EYPG$	Grb2 bound to phosphorylated Tyr_{1138} on ErbB1	nM
$EYPN$	IRS-1 bound to phosphorylated Tyr_{998} on ErbB1	nM
$EYPS$	Shc bound to phosphorylated Tyr_{1172} on ErbB1	nM
$EYPSP$	Phosphorylated Shc bound to pTyr_{1172} on ErbB1	nM
$EYPSPG$	Grb2 bound to phosphorylated Shc bound to ErbB1	nM
$EYPSPGS$	Sos bound to ErbB1-bound Grb2-P-Shc complex	nM
$EYPSPGSaR$	RasGTP bound to allosteric site on Sos-Grb2-ShcP-ErbB1 complex	nM
$EYPSPGSaRcR$	RasGTP and RasGDP bound to Sos-Grb2-ShcP-ErbB1 complex	nM
$EYPSPGScR$	RasGDP bound to catalytic site on Sos-Grb2-ShcP-ErbB1 complex	nM
$EYPGS$	Sos bound to Grb2 on ErbB1	nM
$EYPGSaR$	RasGTP bound to allosteric site on Sos-Grb2-ErbB1 complex	nM
$EYPGSaRcR$	RasGTP and RasGDP bound to Sos-Grb2-ErbB1 complex	nM
$EYPGScR$	RasGDP bound to catalytic site on Sos-Grb2-ErbB1 complex	nM
$EYPT$	PTP bound to phosphorylated Tyr_{1016} on ErbB1	nM
$EYPPL$	PLC-γ bound to phosphorylated Tyr_{998} on ErbB1	nM
$EYPPLP$	Phosphorylated PLC-γ bound to phosphorylated Tyr_{998} on ErbB1	nM
SPG	Grb2 bound to phosphorylated Shc in cytosol	nM
PLP	Phosphorylated PLC-γ in cytosol	nM
$PLPI$	Phosphorylated PLC-γ bound to cytoskeleton	nM
RsT	Active Ras-GTP in cytosol	nM
RsD	Unactive Ras-GDP in cytosol	nM
GS	Sos bound to Grb2 in cytosol	nM

Table S2 - Rate laws for reaction network. Reaction rate equations of the mathematical model for early ErbB1 signaling events.

Rxn No.	Rate Relationship
	Reaction class 0: Receptor synthesis
R0	$= r_{syn}$
	Reaction class 1: Degradation of unactive receptor
R1a	$= -k_{deg1} \cdot E1$
R1b	$= -k_{deg1} \cdot EE1$
R1c	$= -k_{deg1} \cdot E_{11}$
	Reaction class 2: Ligand binding
R2	$= k_{f2} \cdot E \cdot E_1 - k_{r2} \cdot EE_1$
	Reaction class 3: Dimerization of ligand-bound ErbB1
R3	$= k_{f3} \cdot EE_1^2 - k_{r3} \cdot E_{11}$
	Reaction class 4: ErbB1 phosphorylation and dephosphorylation
R4	$= k_{f4} \cdot E_{11} - k_{PTP} \cdot EYPT \cdot E11P$
	Reaction class 5: Degradation of phosphorylated ErbB1
R5	$= k_{deg5} \cdot E_{11}P$
	Reaction class 6: Grb2 binding to phosphorylated ErbB1
R6	$= 2 \cdot k_{f6} \cdot EYP \cdot G - k_{r6} \cdot EYPG$
	Reaction class 7: Irs-1 binding to phosphorylated ErbB1
R7	$= 2 \cdot k_{f7} \cdot EYP \cdot N - k_{r7} \cdot EYPN$
	Reaction class 8: Shc binding to phosphorylated ErbB1
R8	$= 6 \cdot k_{f8} \cdot EYP \cdot Sh - k_{r8} \cdot EYPS$
R10	$= k_{r8} \cdot EYPSP - 6 \cdot k_{f8} \cdot EYP \cdot ShP$
	Reaction class 9: Phosphorylation of ErbB1-bound Shc
R9	$= k_{f9} \cdot EYPS - k_{PTP} \cdot EYPSP \cdot EYPT$
	Reaction class 10: Shc-P dephosphorylation in cytosol
R11	$= k_{r11} \cdot ShP$

Table S2 - Rate laws for reaction network - continued.

Rxn No.	Rate Relationship
	Reaction class 11: Grb2 binding to ErbB1-bound Shc-P
R12	$= k_{f12} \cdot EYPSP \cdot G - k_{r12} \cdot EYPSPG$
	Reaction class 12: Sos binding to ErbB1-bound Grb2
R13	$= k_{f13} \cdot EYPSPG \cdot S - k_{r13} \cdot EYPSPGS$
R14	$= k_{f13} \cdot EYPG \cdot S - k_{r13} \cdot EYPGS$
	Reaction class 13: PTP binding to ErbB1 Tyr_{1110}
R15	$= k_{f15} \cdot EYP \cdot T - k_{r15} \cdot EYPT$
	Reaction class 14 - Shc-P-Grb2 complex binding to phosphorylated ErbB1
R16	$= 6 \cdot k_{f16} \cdot EYP \cdot SPG - k_{r16} \cdot EYPSPG$
	Reaction class 15 - Grb2 binding to Shc-P in cytosol
R17	$= k_{f17} \cdot ShP \cdot G - k_{r17} \cdot SPG$
	Reaction class 16 - PLC-γ binding to phosphorylated ErbB1
R18	$= k_{f18} \cdot EYP \cdot PL - k_{r18} \cdot EYPPL$
	Reaction class 17 - Phosphorylation of ErbB1-bound PLC-γ
R19	$= k_{f19} \cdot EYPPL - k_{PTP} \cdot EYPPLP \cdot EYPT$
	Reaction class 18 - Dissociation of phosphorylated PLC-γ-P from ErbB1
R20	$= k_{r20} \cdot EYPPLP - k_{f20} \cdot EYP \cdot PLP$
	Reaction class 19 - Dephosphorylation of PLC-γ-P in cytosol
R21	$= k_{r21} \cdot PLP$
	Reaction class 20 - Sequestering of PLC-γ-P by binding to cytoskeletal structures
R22	$= k_{f22} \cdot PLP - k_{r22} \cdot PLPI$
	Reaction class 21 - RasGTP binding to allosteric site on ErbB1-bound Sos
R23a	$= k_{f23} \cdot RsT \cdot EYPSPGS - k_{r23} \cdot EYPSPGSaR$
R23b	$= k_{f23} \cdot RsT \cdot EYPGS - k_{r23} \cdot EYPGSaR$
R23c	$= k_{f23} \cdot RsT \cdot EYPSPGScR - k_{r23} \cdot EYPSPGSaRcR$
R23d	$= k_{f23} \cdot RsT \cdot EYPGScR - k_{r23} \cdot EYPGSaRcR$

Table S2 - Rate laws for reaction network - continued.

Rxn No.	Rate Relationship
	Reaction class 22 - RasGDP binding to ErbB1-bound Sos
R24a	$= k_{f27} \cdot RsD \cdot EYPSPGS - k_{r27} \cdot EYPSPGScR$
R24b	$= k_{f27} \cdot RsD \cdot EYPGS - k_{r27} \cdot EYPGScR$
R24c	$= k_{f27} \cdot RsD \cdot EYPSPGSaR - k_{r27} \cdot EYPSPGSaRcR$
R24d	$= k_{f27} \cdot RsD \cdot EYPGSaR - k_{r27} \cdot EYPGSaRcR$
	Reaction class 23 - RasGDP to RasGTP by allosteric-bound ErbB1-bound Sos
R25a	$= k_{cat29} \cdot EYPSPGSaRcR$
R25b	$= k_{cat29} \cdot EYPGSaRcR$
	Reaction class 24 - RasGDP to RasGTP by ErbB1-bound Sos
R26a	$= k_{cat31} \cdot EYPSPGScR$
R26b	$= k_{cat31} \cdot EYPGScR$
	Reaction class 25 - RasGTP hydrolysis to RasGDP in cytosol
R27	$= k_{f33} \cdot RsT$
	Reaction class 26 - Grb2 association with Sos in cytosol
R28	$= k_{f36} \cdot G \cdot S - k_{r36} \cdot GS$
	Reaction class 27 - Grb2-Sos complex association with phosphorylated ErbB1
R29	$= 2 \cdot k_{f37} \cdot GS \cdot EYP - k_{r37} \cdot EYPGS$
	Reaction class 28 - Grb2-Sos complex association with ErbB1-bound Shc-P
R30	$= k_{f38} \cdot GS \cdot EYPSP - k_{r38} \cdot EYPSPGS$
	Reaction class 29 - Add EGF to cell culture
R31	$= 2.5 * (erf(2 * (t - 1.0sec)) - erf(2 * (t - 5.0sec)))$

Table S3 - List of model parameters. The values for the reaction rate parameters correspond to the expectation maximum values obtained from the three AMCMC. The Gelman-Rubin potential scale reduction factor (PSRF) was estimated from the three AMCMC chains.

Symbol	Definition	Value	Units	Refs	PSRF
r_{syn}	Receptor synthesis rate	1.55×10^{-3}	nM sec^{-1}	[1]	–
k_{deg1}	Unbound ErbB1 receptor degradation	1.55×10^{-5}	sec^{-1}	–	–
k_{f2}	Binding of EGF ligand to ErbB1	8.60×10^{-3}	nM^{-1} sec^{-1}	[2]	–
k_{r2}	Dissociation of EGF ligand from ErbB1 - K_D = 0.20 nM	1.72×10^{-3}	sec^{-1}	[2]	–
k_{f3}	Dimerization of ligand-bound ErbB1	6.91×10^{3}	nM^{-1} sec^{-1}	*	2.36
k_{r3}	Dissociation of ligand-bound ErbB1 dimer - K_D = 10 nM	6.91×10^{4}	sec^{-1}	[3]	–
k_{f4}	Phosphorylation of ligand-bound ErbB1 dimers	1.37×10^{4}	sec^{-1}	*	1.44
k_{PTP}	Dephosphorylation mediated by PTP-ErbB1 complex	1.82×10^{5}	sec^{-1}	*	1.22
k_{deg5}	Effective phosphorylated ErbB1 degradation	2.32×10^{-4}	sec^{-1}	*	1.55
k_{f6}	Grb2 association with ErbB1 phosphotyrosine	2.19×10^{4}	nM^{-1} sec^{-1}	*	2.83
k_{r6}	Dissociation of Grb2 from phosphorylated Tyr_{1138} - K_D = 724 nM	1.59×10^{7}	sec^{-1}	[4]	–
k_{f7}	Irs-1 association with ErbB1 phosphotyrosine	0.100	nM^{-1} sec^{-1}	–	–
k_{r7}	Dissociation of Irs-1 from phosphorylated Tyr_{1172} - K_D = 1282 nM	128.2	sec^{-1}	[4]	–
k_{f8}	Shc association with ErbB1 phosphotyrosine	43.3	nM^{-1} sec^{-1}	*	1.44
k_{r8}	Dissociation of Shc from phosphorylated Tyr_{1172} - K_D = 78 nM	3.38×10^{3}	sec^{-1}	[5]	–
k_{f9}	Shc phosphorylation	3.58×10^{7}	nM^{-1} sec^{-1}	*	1.79
k_{r11}	Free Shc dephosphorylation in cytosol	818	sec^{-1}	*	1.76
k_{f12}	Grb2 association with P-Shc-ErbB1 complex	1.19×10^{6}	nM^{-1} sec^{-1}	*	1.77
k_{r12}	Dissociation of Grb2 from P-Shc-ErbB1 complex - K_D = 151 nM	1.80×10^{8}	sec^{-1}	[6]	–
k_{f13}	Sos association with Grb2-P-Shc-ErbB1 complex	2.81×10^{4}	nM^{-1} sec^{-1}	*	1.58
k_{r13}	Dissociation of Sos from Grb2-P-Shc-ErbB1 complex - K_D = 117 nM	3.29×10^{6}	sec^{-1}	Thermo	–
k_{f15}	PTP association with phosphorylated ErbB1	2.46×10^{-5}	nM^{-1} sec^{-1}	*	1.2
k_{r15}	Dissociation of PTP from phosphorylated Tyr_{1016} ErbB1 - K_D = 379 nM	9.31×10^{-3}	sec^{-1}	[4]	–
$VeVc$	Ratio of the water volume in the cytoplasm relative to extracellular medium per cell	333.3	–	[7]	–

* = determined in this study
Thermo = Thermodynamic cycle constraints

Table S3 - List of model parameters - continued.

Symbol	Definition	Value	Units	Refs	PSRF
k_{f16}	ShcP-Grb2 association with ErbB1 phosphotyrosine	1.29×10^5	nM^{-1} sec^{-1}	*	3.76
k_{r16}	Dissociation of ShcP-Grb2 from phosphorylated Tyr_{1172} - K_D = 78 nM	1.00×10^7	sec^{-1}	Thermo	–
k_{f17}	Grb2 association with P-Shc in cytosol	0.330	nM^{-1} sec^{-1}	*	2.96
k_{r17}	Dissociation of Grb2 from P-Shc - K_D = 151 nM	4.98×10^1	sec^{-1}	Thermo	–
k_{f18}	PLC-γ association with ErbB1 phosphotyrosine	1.50×10^4	nM^{-1} sec^{-1}	*	2.56
k_{r18}	Dissociation of PLC-γ from phosphorylated Tyr_{1016} - K_D = 794 nM	1.19×10^7	sec^{-1}	[5]	–
k_{f19}	Phosphorylation of PLC-γ bound to ErbB1	1.36×10^4	sec^{-1}	*	4.71
k_{f20}	PLC-γ-P association with ErbB1 phosphotyrosine	8.92×10^{-2}	nM^{-1} sec^{-1}	*	2.37
k_{r20}	Dissociation of PLC-γ-P from phosphorylated Tyr_{1016} - K_D = 794 nM	71.2	sec^{-1}	[4]	–
k_{r21}	Dephosphorylation of PLC-γ-P in cytosol	72.9	sec^{-1}	*	3.29
k_{f22}	PLC-γ-P association with cytoskeleton	83.2	nM^{-1} sec^{-1}	*	4.07
k_{r22}	Dissociation of PLC-γ-P from cytoskeleton - K_D = 3.23×10^{-3} nM	0.269	sec^{-1}	*	3.3
k_{f23}	RasGTP binding to Sos allosteric site	1.68×10^5	nM^{-1} sec^{-1}	*	4.48
k_{r23}	Dissociation of RasGTP from Sos allosteric site - K_D = 5280 nM	8.87×10^8	sec^{-1}	*	1.30
k_{f27}	RasGDP binding to Sos catalytic site	2.84×10^{-3}	nM^{-1} sec^{-1}	*	5.86
k_{r27}	Dissociation of RasGDP from Sos catalytic site - K_D = 1.40×10^{-5} nM	3.98×10^{-8}	sec^{-1}	*	3.97
k_{cat29}	RasGDP activation by allosteric-bound membrane Sos	9.70	sec^{-1}	*	1.54
k_{cat31}	RasGDP activation by membrane Sos	0.216	nM^{-1} sec^{-1}	[8]	–
k_{f33}	RasGTP hydrolysis to RasGDP	2.04×10^{-2}	sec^{-1}	*	4.79
k_{f36}	Grb2 association with Sos in cytosol	2.04×10^{-5}	nM^{-1} sec^{-1}	*	5.17
k_{r36}	Dissociation of Grb2 from Sos - K_D = 400 nM	8.16×10^{-3}	sec^{-1}	[6]	–
k_{f37}	Grb2-Sos association with ErbB1 phosphotyrosine	3.55×10^6	nM^{-1} sec^{-1}	*	3.95
k_{r37}	Dissociation of Grb2-Sos from phosphorylated Tyr_{1138} - K_D = 211 nM	7.48×10^8	sec^{-1}	[6]	–
k_{f38}	Grb2-Sos association with P-Shc-ErbB1	2.06×10^{-3}	nM^{-1} sec^{-1}	*	3.45
k_{r38}	Dissociation of Grb2-Sos from P-Shc-ErbB1 - K_D = 44 nM	9.08×10^{-2}	sec^{-1}	[6]	–

Table S3 - List of model parameters - continued.

Symbol	Definition	Value	Units	Refs	PSRF
E_{10}	Initial expression of ErbB1	100	nM	[7]	–
G_0	Initial expression of Grb2	292	nM	*	1.26
N_0	Initial expression of Irs-1	10	nM	–	–
PL_0	Initial expression of PLC-γ	4.37×10^{-3}	nM	*	2.3
Sh_0	Initial expression of Shc	536	nM	*	1.44
S_0	Initial expression of Sos	86.9	nM	*	3.65
T_0	Initial expression of PTP	100	nM	–	–
RsT_0	Initial expression of RasGTP	6.00×10^{-2}	nM	*	6.00
RsD_0	Initial expression of RasGDP	9.48×10^{3}	nM	*	5.64

Table S4 - Differential and algebraic equations that define the mathematical model for early ErbB1 signaling events.

Differential Equations

$d[E_1]/dt = R0 - R1a - R2$
$d[EE_1]/dt = R2 - 2 \cdot R3 - R1b$
$d[E_{11}]/dt = R3 - R4 - R1c$
$d[E_{11}P]/dt = R4 - R5$
$d[G]/dt = -R6 - R12 - R17 - R28$
$d[N]/dt = -R7$
$d[PL]/dt = -R18 + R21$
$d[Sh]/dt = -R8 + R11$
$d[S]/dt = -R13 - R14 - R28$
$d[T]/dt = -R15$
$d[EYPG]/dt = R6 - R14$
$d[EYPN]/dt = R7$
$d[EYPS]/dt = R8 - R9$
$d[EYPSPG]/dt = R12 - R13 + R16$
$d[EYPSPGS]/dt = R13 - R23a - R24a + R26a + R30$
$d[RsT]/dt = -R23a - R23b - R23c - R23d + R25a + R25b + R26a + R26b - R27$
$d[EYPSPGSaRcR]/dt = R23c + R24c - R25a$
$d[E]/dt = R31 - R2/VeVc$
$d[EYPSPGSaR]/dt = R23a - R24c + R25a$
$d[EYPGSaR]/dt = R23b - R24d + R25b$
$d[EYPGSaRcR]/dt = R23d + R24d - R25b$
$d[EYPGScR]/dt = -R23d + R24b - R26b$
$d[EYPT]/dt = R15$
$d[EYPPL]/dt = R18 - R19$
$d[EYPPLP]/dt = R19 - R20$
$d[ShP]/dt = R10 - R11 - R17$
$d[SPG]/dt = -R16 + R17$
$d[PLP]/dt = R20 - R21 - R22$
$d[PLPI]/dt = R22$
$d[EYPSP]/dt = R9 - R10 - R12 - R30$
$d[GS]/dt = R28 - R29 - R30$
$d[EYPGS]/dt = R14 - R23b - R24b + R26b + R29$
$d[RsD]/dt = -R24a - R24b - R24c - R24d + R27$
$d[EYPSPGScR]/dt = -R23c + R24a - R26a$

Algebraic Equation

$$EYP = 2 \cdot E_{11}P - EYPG - EYPN - EYPS - EYPT - EYPSP - EYPSPG - EYPGS - EYPSPGS - EYPSPGSaR - EYPSPGSaRcR - EYPSPGScR - EYPGSaR - EYPGSaRcR - EYPGScR - EYPPL - EYPPLP$$

Table S5 - Binding sites on ErbB1. Equilibrium dissociation constants (K_D in nM) and reaction pathway degeneracy (RPD) for the interaction of Shc, PLC-γ, Grb2, PTPN11, and Irs-1 with specific tyrosine residues of ErbB1 ([4, 5]). The reaction path degeneracy corresponds to the number of binding that exhibit measurable affinity.

Signaling Protein	Phospho-tyrosine residue of ErbB1						RPD
	p-Tyr_{998}	p-Tyr_{1016}	p-Tyr_{1110}	p-Tyr_{1138}	p-Tyr_{1172}	p-Tyr_{1192}	
Grb2	–	–	>2000	724[b]	–	–	2
Irs-1	1500	–	–	–	1282[b]	–	2
Shc[a]	1152	628	321	173	78[b]	337	6
PLC-γ	–	794[b]	–	–	–	–	1
PTPN11	–	379[b]	–	–	–	–	1

[a] PTB-domain binding site
[b] assumed equilibrium binding site

References

[1] Resat H, Ewald JA, Dixon DA, Wiley HS: **An Integrated Model of Epidermal Growth Factor Receptor Trafficking and Signal Transduction**. *Biophys J* 2003, **85**:730–743.

[2] Wilkinson JC, Stein RA, Guyer CA, Beechem JM, Staros JV: **Real-time kinetics of ligand/cell surface receptor interactions in living cells: Binding of epidermal growth factor to the epidermal growth factor receptor**. *Biochemistry* 2001, **40**(34):10230–10242.

[3] Birtwistle MR, Hatakeyama M, Yumoto N, Ogunnaike BA, Hoek JB, Kholodenko BN: **Ligand-dependent responses of the ErbB signaling network: experimental and modeling analyses**. *Mol Sys Bio* 2007, **3**:144.

[4] Jones RB, Gordus A, Krall JA, MacBeath G: **A quantitative protein interaction network for the ErbB receptors using protein microarrays**. *Nature* 2006, **439**:168–174.

[5] Kaushansky A, Gordus A, Chang B, Rush J, MacBeath G: **A quantitative study of the recruitment potential of all intracellular tyrosine residues on EGFR, FGFR1 and IGF1R**. *Molecular Biosystems* 2008, **4**(6):643–653.

[6] Chook YM, Gish GD, Kay CM, Pai EF, Pawson T: **The Grb2-mSos1 complex binds phosphopeptides with higher affinity than Grb2**. *J Biol Chem* 1996, **271**(48):30472–30478.

[7] Kholodenko BN, Demin OV, Moehren G, Hoek JB: **Quantification of Short Term Signaling by the Epidermal Growth Factor Receptor**. *J Biol Chem* 1999, **274**:30169–30181.

[8] Gureasko J, Galush WJ, Boykevisch S, Sondermann H, Bar-Sagi D, Groves JT, Kuriyan J: **Membrane-dependent signal integration by the Ras activator Son of sevenless**. *Nature Struct Mol Bio* 2008, **15**(5):452–461.
